# Urgent Support Is Needed for More Tinnitus Research

**DOI:** 10.2188/jea.JE20240427

**Published:** 2025-08-05

**Authors:** Carlotta M. Jarach, Jorge P. Simoes, Winfried Schlee, Berthold Langguth, Silvano Gallus

**Affiliations:** 1 Department of Medical Epidemiology, Istituto di Ricerche Farmacologiche Mario Negri IRCCS, Milan, Italy; 2 Department of Psychology, Health and Technology, University of Twente, Enschede, The Netherlands; 3 Institute for Information and Process Management, Eastern Switzerland University of Applied Sciences, St. Gallen, Switzerland; 4 Department of Psychiatry and Psychotherapy, University of Regensburg, Regensburg, Germany

**Keywords:** tinnitus, epidemiology, global burden, ageing, disability

Tinnitus is a major public health condition, described as the sensation of hearing sounds in the absence of external stimuli^1^; its global burden can be compared to other significant disabilities, such as migraine and pain,^2^ with over 740 million people having experienced at least one episode of tinnitus in their lifetime. More importantly, 120 million people suffer from severe forms that greatly impair their quality of life,^2^ even increasing the likelihood of suicide.^3^ This condition also places a significant financial burden on people with tinnitus, who may spend up to 1,500 euros annually out-of-pocket^4^ on treating a symptom for which there is currently no definitive cure.^1^

Globally, we are witnessing a significant shift in disease burden towards an increasingly prominent presence of non-communicable diseases. As life expectancy rises and the prevalence of tinnitus increases with age,^4^ a rise in the number of people with tinnitus can be expected in the coming years.

Tinnitus prevalence tends to increase^5^; but even if it stays stable, according to official international population projections, and assuming as constant the available age-specific tinnitus prevalence estimates,^2^ we can anticipate a 57% increase in the adult population with severe tinnitus in 2050 compared to 2024. This increase is attributed to the overall growth of the global adult population and the aging phenomenon. As a result, the number of adults with severe tinnitus is expected to rise from 117 million in 2024 to 183 million in 2050 (Table 1).

**Table 1.** Projections of the global adult population (in thousands) with any tinnitus and severe tinnitus in 2050 compared to 2024

	Global adult population^a^		People with any tinnitus			People with severe tinnitus		
		
	2024	2050	Prevalence,^b^ %	2024	2050	Prevalence,^b^ %	2024	2050
Age group, years								

18–44	3,204,790	3,556,955	9.7%	310,865	345,025	0.4%	12,819	14,228
45–64	1,710,780	2,223,443	13.7%	234,377	304,612	2.7%	46,191	60,033
≥65	833,481	1,578,015	23.6%	196,702	372,412	6.9%	57,510	108,883

Total	5,749,051	7,358,413		741,943	1,022,048		116,520	183,144

Change between 2024 and 2050, %		+28%			+38%			+57%

^a^Population estimates were based on the 2024 Revision of World Population Prospects,^6^ with constant-fertility variant. Source: United Nations Department of Economic and Social Affairs Population Division. World Population Prospects 2024. Available from: http://population.un.org/wpp/. Under the constant-fertility scenario, fertility in all countries remain constant at the level projected for 2024. Mortality and migration assumptions are the same as those in the medium fertility scenario. For more details on the statistical assumptions, refer to UN World Population Prospects 2024: Methodology of the United Nations population estimates and projections (UN DESA/POP/2024/DC/NO. 10).

^b^Prevalence estimates of any tinnitus and severe tinnitus were based on Jarach et al, JAMA Neurol. 2022; 79(9): 888–900.^2^

In recent years, research on tinnitus has increased, but funding remains scarce, as it has been over the past decade.^1^ Given the challenges and the far-reaching impacts of an aging population, from individual and community burdens to broader socioeconomic consequences, within healthcare and beyond, it is increasingly urgent to raise awareness among policymakers and the medical research community overall about the necessity of funding research on tinnitus, both to better prevent its incidence and worsening, as well as to develop effective personalized treatments.
